# Remotely acting *SMCHD1* gene regulatory elements: in silico prediction and identification of potential regulatory variants in patients with FSHD

**DOI:** 10.1186/s40246-015-0047-x

**Published:** 2015-10-07

**Authors:** Mary B. Mayes, Taniesha Morgan, Jincy Winston, Daniel S. Buxton, Mihir Anant Kamat, Debbie Smith, Maggie Williams, Rebecca L. Martin, Dirk A. Kleinjan, David N. Cooper, Meena Upadhyaya, Nadia Chuzhanova

**Affiliations:** School of Science and Technology, Nottingham Trent University, Clifton Lane, Nottingham, NG11 8NS UK; Institute of Medical Genetics, School of Medicine, Cardiff University, Heath Park, Cardiff, CF14 4XN UK; Blood Sciences Department and Bristol Genetics Laboratory, Southmead Hospital, Westbury-on-Trym, Bristol BS10 5NB UK; MRC Human Genetics Unit, Institute of Genetics and Molecular Medicine, University of Edinburgh, Edinburgh, EH4 2XU UK; Present address: Cardiovascular Epidemiology Unit, Department of Public Health and Primary Care, University of Cambridge, Cambridge, CB1 8RN UK

**Keywords:** Regulatory mutations, Regulatory elements, Hi-C data, *SMCHD1*, FSHD

## Abstract

**Background:**

Facioscapulohumeral dystrophy (FSHD) is commonly associated with contraction of the D4Z4 macro-satellite repeat on chromosome 4q35 (FSHD1) or mutations in the *SMCHD1* gene (FSHD2). Recent studies have shown that the clinical manifestation of FSHD1 can be modified by mutations in the *SMCHD1* gene within a given family. The absence of either D4Z4 contraction or *SMCHD1* mutations in a small cohort of patients suggests that the disease could also be due to disruption of gene regulation. In this study, we postulated that mutations responsible for exerting a modifier effect on FSHD might reside within remotely acting regulatory elements that have the potential to interact at a distance with their cognate gene promoter via chromatin looping. To explore this postulate, genome-wide Hi-C data were used to identify genomic fragments displaying the strongest interaction with the *SMCHD1* gene. These fragments were then narrowed down to shorter regions using ENCODE and FANTOM data on transcription factor binding sites and epigenetic marks characteristic of promoters, enhancers and silencers.

**Results:**

We identified two regions, located respectively ~14 and ~85 kb upstream of the *SMCHD1* gene, which were then sequenced in 229 FSHD/FSHD-like patients (200 with D4Z4 repeat units <11). Three heterozygous sequence variants were found ~14 kb upstream of the *SMCHD1* gene. One of these variants was found to be of potential functional significance based on DNA methylation analysis. Further functional ascertainment will be required in order to establish the clinical/functional significance of the variants found.

**Conclusions:**

In this study, we propose an improved approach to predict the possible locations of remotely acting regulatory elements that might influence the transcriptional regulation of their associated gene(s). It represents a new way to screen for disease-relevant mutations beyond the immediate vicinity of the specific disease gene. It promises to be useful for investigating disorders in which mutations could occur in remotely acting regulatory elements.

**Electronic supplementary material:**

The online version of this article (doi:10.1186/s40246-015-0047-x) contains supplementary material, which is available to authorized users.

## Background

Facioscapulohumeral muscular dystrophy (FSHD) is the most prevalent of the nine primary types of muscular dystrophy affecting adults and children; it is characterized by the weakness and atrophy of the facial and shoulder girdle muscle extending to the abdominal and lower limb muscle [1]. Two genetic loci are associated with the disease. The FSHD1 locus maps to 4q35 [2] and accounts for 95 % of clinical diseases in an FSHD context [1, 3]. A second FSHD locus, FSHD2, exists and is phenotypically indistinguishable from FSHD1 [2]. FSHD1 patients harbour a large deletion in the polymorphic *D4Z4* macro-satellite repeat array at 4q35 and invariably present with 1–10 repeats whereas non-affected individuals possess 11–150 repeats. Each 3.3-kb *D4Z4* unit contains a double homeobox 4 (*DUX4*) gene that, among others, is transcriptionally activated on contraction of the 4q35 repeat array as a consequence of the induction of chromatin remodelling of the 4qter region. A number of 4q subtelomeric sequence variants are now recognized, although FSHD1 only occurs in association with ‘permissive’ haplotypes, each of which is associated with a polyadenylation signal located immediately distal of the last *D4Z4* unit [4]. Approximately 5 % of FSHD patients lack a contraction of the *D4Z4* array, and the disease aetiology has been ascribed to a putative FSHD2 locus. Whole-exome sequencing identified the *SMCHD1* gene as the causative agent at the FSHD2 locus [5]. In FSHD2 families, the disease exhibits a more complex digenic inheritance because mutations in the chromosome-18-located *SMCHD1* gene segregate independently from the FSHD-permissive 4q haplotype [5]. *SMCHD1* is a member of a condensing/cohesion family of chromatin compact complexes that bind to the D4Z4 array [6]. However, not all FSHD2 patients can be explained by the lack of contraction of the *D4Z4* repeats or mutations in the *SMCHD1* gene, suggesting either that mutations may reside within the *SMCHD1* non-coding region or that the cause of FSHD in these families could be linked to yet another FSHD locus. Although FSHD1 and FSHD2 are characterized by different underlying genetic defects, they both appear to be caused by transcriptional de-repression of *DUX4* in the skeletal muscle [7].

DNA methylation changes the conformation of the chromatin and hence the accessibility of the encoded gene(s) in the vicinity. Hypermethylation generally leads to the compaction of chromatin, thereby reducing gene expression. Conversely, hypomethylation generally serves to relax the chromatin thereby upregulating gene expression. FSHD-affected individuals display hypomethylation at D4Z4 units, whereas unaffected subjects exhibit hypermethylation whilst FSHD non-manifesting carriers have an intermediate level of methylation [8]. The specific loss of histone H3 lysine 9 trimethylation and HP1 gamma/cohesion binding at D4Z4 repeats is reported to be associated with FSHD [6].

Recent studies have reported that the clinical manifestation of FSHD1 can be modified by mutations in the *SMCHD1* gene within a given family [9–11] although pathogenic FSHD mutations remained undetected in a majority of FSHD2 patients. The undetected mutations in such patients could, in principle, reside within regulatory elements, possibly at some distance from the *SMCHD1* gene, disrupting regulation of the gene.

Advances in techniques for capturing three-dimensional chromosome conformations (3C) have been prompted by the view that direct long-range interactions occur between gene promoters and distal genomic regions, bringing them into close spatial proximity through looping interactions [12], thereby explaining the impact of pathological mutations that are known to occur at some considerable distance from the genes whose function they influence [13]. Genome-wide mapping of long-range interactions using the Hi-C [14], the recently proposed in situ Hi-C [15] and Capture Hi-C [16] methods has made it possible to assess the propensity of genomic regions to form looping interactions by surveying their interaction frequencies; the latter two methods have yielded interaction maps of sufficiently high resolution to be practically useful in this regard.

Studies of the long-range interactions of ~22,000 promoters in two human blood cell lines, including lymphoblastoid cell line GM12878, employing the Capture Hi-C method [16] have revealed that interacting fragments are enriched in DNaseI hypersensitive sites (DHSs), a classical marker of regulatory regions [17]; the H3K4me3 histone mark, the tri-methylation of histone H3 at lysine 4, mainly associated with promoters that are active or poised to be activated ([18], reviewed in [19]); the H3K4me1 histone mark, the mono-methylation of histone H3 at lysine 4, a well-established feature of enhancers and promoters; and the H3K27ac mark, the acetylation of histone H3 at lysine 27, characteristic of active enhancers ([18], reviewed in [19]).

In this study, we postulated that mutations responsible for the clinical manifestation of FSHD or playing a disease-modifier role might reside within remotely acting regulatory elements that have the potential to interact with the DNA fragment containing the *SMCHD1* gene and that these elements might be located within regions enriched in epigenetic features characteristic of regulatory regions. We identified two remotely located putative regulatory elements residing within regions respectively strongly and weakly interacting with the fragment containing the *SMCHD1* gene. We screened these in silico-predicted regulatory regions for mutations in 229 FSHD patients (200 patients exhibiting <11 D4Z4 repeat units and 29 patients >11 D4Z4 repeat units). We report two novel sequence variants in a putative control region proximal to the *SMCHD1* gene (~14 kb upstream of the gene) in two FSHD families.

## Results and discussion

### In silico prediction of interacting fragments and potential remotely acting regulatory regions of *SMCHD1*

**Fig. 1:**
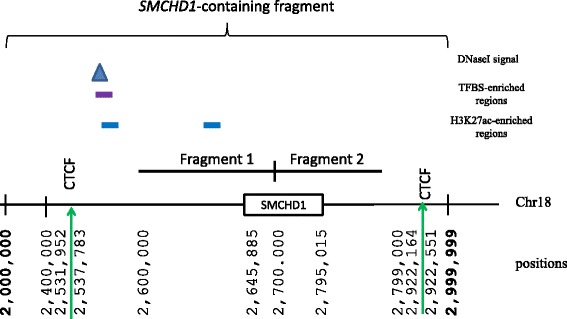
Schematic representation of the chromosomal regions enriched in transcription factor binding sites (TFBS), histone mark H3K27ac and DNAseI hypersensitive signal within 1-Mb-long *SMCHD1*-containing DNA fragments (not to scale). Two consecutive 100-kb fragments harbouring the *SMCHD1* gene are denoted as *Fragments 1* and *2*. CTCF is a binding site for the insulator protein

Hi-C data, available at www.ncbi.nlm.nih.gov/geo/ (accession number GSE18199), indicated that a 1-Mb fragment of chromosome 18, starting at position 2,000,000 and ending at position 2,999,999, which we have termed the *SMCHD1*-containing fragment since it contains the entire *SMCHD1* gene (positions 2,645,885–2,795,015; hg18), interacts most strongly with itself (3371 intra-chromosomal interactions with the *SMCHD1*-containing fragment were also recorded in Hi-C data) and with the adjacent 1-Mb downstream fragment on chromosome 18 (positions 3,000,000–3,999,999; 663 interactions). On a 100-kb scale, the *SMCHD1* gene was found to occupy two consecutive fragments (fragment 1: 2,600,000–2,699,999 and fragment 2: 2,700,000–2,799,999, Fig. 1); for each of these fragments, the number of intra-chromosomal interactions was also available from the Hi-C data. Fragment 1 and fragment 2 interacted most strongly with themselves (256 and 237 interactions, respectively) whereas the number of interactions with other 100-kb fragments on chromosome 18 did not exceed 30. The ENCODE data indicated the presence of two weak CTCF-binding sites (scores <445/1000) between positions 2,531,952 and 2,537,783 and a strong one (with score 719/1000) at positions 2,922,164 and 2,922,551. Among other functions, CTCF and its associated proteins that bind to the CTCF-binding sites are thought to play a role in forming barriers between regulatory regions but are also involved (together with other transcription factors) in controlling chromatin-looping interactions and structure (reviewed in [19]) although a recent study [16] indicated that insulator activities of CTCF could be tissue- or cell-line-specific. Because the first CTCF-binding site was found within the 100-kb region (2,500,000–2,599,999), which has only 23 interactions with the *SMCHD1*-containing region, we opted to extend the search for potential regulatory elements to positions 2,531,952–2,922,551.

The ENCODE data on histone modification marks, H3K4me1 and H3K27ac, available for eight cell lines, reveal the presence of these marks in the GM12878 cell line between positions 2,624,475 and 2,635,775. The ENCODE data also indicated the presence of an H3K4me3-enriched region, characteristic of an active promoter, somewhere between positions 2,644,486 and 2,648,785 on the interacting fragment (Fig. 1). There is a strong enhancer at positions 2,631,000–2,632,200 and a transcription factor binding site (TFBS)-enriched region between positions 2,631,527 and 2,632,188; the latter region, ~14 kb upstream of the *SMCHD1* gene, was selected for sequencing in FSHD patients.

A second region, distal (~84 kb) to the *SMCHD1* gene, was also selected for sequencing (positions 2,561,489–2,562,509) on the basis that it is enriched in H3K4me3 and TFBSs and corresponds to a peak of DNaseI sensitivity signal, characteristic of open chromatin and an active *cis*-regulatory region; it also shows a weak interaction with the *SMCHD1*-containing region. The ENCODE data on histone modification marks H3K4me1 and H3K27ac revealed the presence of these marks in eight cell lines, the exception being GM12878, between positions 2,557,575 and 2,566,775. ChromHMM track indicated the presence of an active promoter at positions 2,560,200–2,563,800, which corresponds precisely to the *METTL4* gene promoter.

### Search for sequence variants within the potential remotely acting regulatory regions of *SMCHD1*

The two predicted regions, respectively ~14 kb and ~85 kb upstream of the *SMCHD1* gene, were sequenced in 229 FSHD patients of Central European descent (the DNA-sequencing procedure is described in the Additional file 1; the primers used are given in Table S1). DNA sequence data from these regions, derived from the 1000 Genomes Project, are available for 226 individuals from the CEU (Utah Residents with Northern and Western European Ancestry) population and were used as controls.

In addition, DNA from the parents of the FSHD probands, from families 1 and 2, was analysed. Family 1 was referred to us because the proband exhibited facial and shoulder girdle weakness; both parents were healthy. In family 2, the proband had early onset FSHD, the parents of the proband were healthy and non-consanguineous and no other member of the family was affected with FSHD. Neither parent showed any evidence of facial or other muscle weakness.

**Fig. 2:**
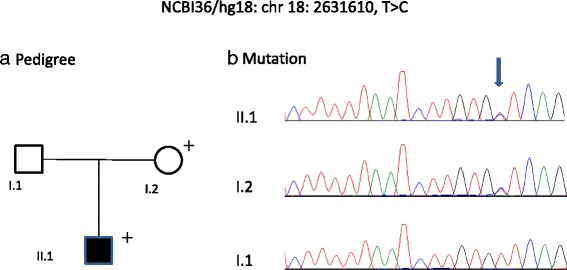
Pedigree (**a**) and mutation (**b**) in the proximal promoter region of the *SMCHD1* gene, family 1

**Fig. 3:**
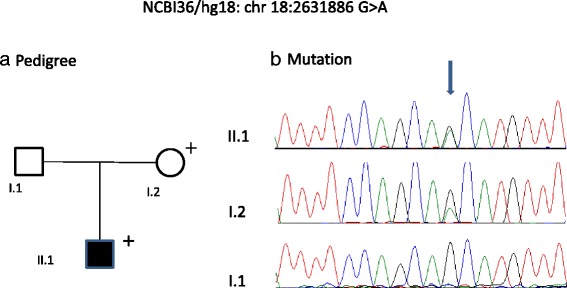
Pedigree (**a**) and mutation (**b**) in the proximal promoter region of the *SMCHD1* gene, family 2

We identified variants in the region proximal (~14 kb upstream) to the *SMCHD1* gene in two probands from families 1 and 2. These variants were not present in dbSNP (http://www.ncbi.nlm.nih.gov/snp) or the 1000 Genomes Project data. The two variants, NCBI36/hg18:chr18:2,631,610 T > C and NCBI36/hg18:chr18:2,631,886 G > A, were found in probands from families 1 and 2, respectively (Figs. 2 and 3). They were also identified in the clinically unaffected mothers of these two patients.

Variant NCBI36/hg18:chr18:2,631,610 T > C was found in the proband who was originally tested due to facial and shoulder girdle muscle weakness. All three members of this family (family 1) were found to harbour >11 D4Z4 repeats, but only the proband was symptomatic. No mutations within the *SMCHD1*-coding region were detected. Both the proband and the unaffected mother harboured the heterozygous sequence change NCBI36/hg18:chr18:2,631,610 T > C. This nucleotide is conserved in primates (human, chimpanzee, gorilla, orang-utan, gibbon, rhesus monkey, crab-eating macaque, baboon, green monkey and squirrel monkey); the evolutionary conservation score, *phyloP* [20], calculated across 44 vertebrates, was 0.557, indicating a conserved nucleotide.

The second novel sequence change, NCBI36/hg18:2,631,886 G > A, was found in the proband from family 2. Molecular testing based on *EcoR*I/*Bln*I analysis [10] identified two intact D4Z4 repeats in the proband and >11 in the mother. Interestingly, his unaffected mother was a mosaic for this change (Fig. 3). This nucleotide is conserved in primates (human, chimpanzee, orang-utan, rhesus monkey, baboon and marmoset). The evolutionary conservation score across 44 vertebrate species was 0.431. As the proband in this family has FSHD1, it is possible that this sequence variant could modify the clinical expression of FSHD1 as this patient exhibited weakness of facial and shoulder girdle muscle with early onset (8 years of age).

The third heterozygous sequence variant was found in the region proximal (~14 kb upstream) to the *SMCHD1* gene at position NCBI36/hg18:2,631,858; a surprisingly high proportion (182/229) of patients were found to have a C-allele at this position with 47/229 patients having a T-allele. A T/C polymorphism at this position is recorded in dbSNP (rs7229070) with C being the minor allele with MAF = 0.30 for a CEU (Utah Residents with Northern and Western European Ancestry) population (226 genomes) sequenced by the 1000 Genomes Project. According to dbSNP, the clinical significance of this SNP is unknown. This polymorphism occurs in a region which is evolutionarily conserved among primates; all the other primate species examined have nucleotide T in this position in their wild-type sequences. In addition, this nucleotide is also conserved in members of other mammalian orders including the artiodactyla, rodentia and carnivora (*phyloP* score across 44 vertebrate species is 0.691).

All three sequence changes, 2,631,610 T > C, 2,631,858 T > C and 2,631,886 G > A, occur in close proximity to, or within, the DNaseI hypersensitive cluster (positions 2,631,700–2,631,930) characterized by a high score (976 out of 1000) that corresponds to an open chromatin domain and rich in TFBSs (IRF1, STAT1, STAT2, RUNX3, BATF, SPI1) with IRF1 (positions 2,631,639–2,632,023), STAT1 (2,631,540–2,632,016), STAT2 (2,631,654–2,632,010) and RUNX3 (2,631,672–2,631,955) transcription factor binding sites having the highest score (1000/1000). All three sequence variants also occur in a region enriched in enhancer-associated marks, H3K4me1 and H3K27ac, observed in cell line GM12878.

Recently published data on looping interactions [15] reveal a strong looping interaction in the GM12878 cell line, between a 5-kb fragment corresponding to the promoter region of the *SMCHD1* gene (positions 2,640,000–2,645,000) and a 5-kb region (positions 2,630,000–2,635,000) harbouring the three variants found (note that all positions were ‘lifted over’ to the hg18 assembly). It was not possible to confirm the existence of a looping interaction between the *SMCHD1* gene promoter and the region ~14 kb upstream of the gene harbouring the variants using Capture Hi-C data [16] since only interactions between promoters and interacting fragments separated by >20 kb were reported.

### Methylation analysis of families 1 and 2

The SMCHD1 protein plays a role in the methylation of large chromosomal regions, including the X chromosome and the D4Z4 array. It is required for the maintenance of X inactivation in females and hypermethylation of CpG islands associated with the inactive X chromosome. Loss of *SMCHD1* activity in FSHD2 patients reduces methylation of the D4Z4 array, allowing the transcriptional machinery to gain access to the *DUX4* gene [5]. FSHD2 is caused by the co-inheritance of two independent events: an FSHD-permissive chromosome 4 haplotype (necessary for the polyadenylation of *DUX4* mRNA) and a variant in *SMCHD1* [5]. SMCHD1 regulates chromatin repression in a wide variety of different organisms. Given the wider role of *SMCHD1* in regulating methylation, it may be that *SMCHD1* serves as a modifier in human genetic disease [9–11].

Hartweck et al. [21] identified an extreme demethylation of region DR1 within the D4Z4 repeat and demonstrated that this region is hypomethylated in the majority of FSHD2 patients. On the basis of this finding, the methylation of this region was measured in blood samples from members of families 1 and 2 using bisulphite conversion followed by pyrosequencing; the ratios of C vs. T at each of the ten statistically validated sites within the DR1 region were averaged (see Additional file 1). The proband from family 2 has two D4Z4 repeat units and exhibits a methylation level of 24 %, compatible with the methylation level seen in FSHD2 patients [5]. The clinically unaffected mother of this patient was a mosaic for the variant, but her D4Z4 repeat was not hypomethylated. Based on a two D4Z4 unit array combined with the 4qA haplotype, hypomethylation and evolutionary conservation, we propose that the promoter variant in *SMCHD1* (2,631,886 G > A) represents a plausible candidate for modifying the expression of FSHD1 in this patient.

The results of methylation analysis for the proband from family 1 were inconclusive; no hypomethylation of D4Z4 was observed in the DR1 region, but other regions (DR2 and DR3) were not tested. Based on an epigenetic signature, Jones et al. [22] have developed a new laboratory test that identifies symptomatic FSHD1 and 2 from non-penetrant FSHD carriers and other muscular dystrophies. In a recent study, Huichalaf et al. [23] have shown that DNA methylation does not correlate with the density of CpG dinucleotides within D4Z4. However, DNA methylation and histone de-acetylation are required to maintain the repression of the FSHD candidate gene. H3K27me3 was reported to be enriched at D4Z4 in healthy subjects and significantly decreased in FSHD patients. Further functional ascertainment will be required in order to establish the pathological significance of the variants identified in our study.

Methylation plays an important role in the marked clinical variability seen in FSHD patients. Recent studies indicate that a combination of genetic and epigenetic factors act on the D4Z4 repeat array to determine the probability of *DUX4* expression in the skeletal muscle and influence disease penetrance and progression [24]. In FSHD patients harbouring 1 to 6 D4Z4 units, the D4Z4 unit number appears to correlate inversely with the severity of the disease, but in patients with 7–10 D4Z4 units, severity depends upon the factors that regulate methylation [8]. In a family with a case of full-blown FSHD and a relative with a non-penetrant mutation bearing an identical number of D4Z4 units, the difference in clinical severity between these two family members appears to be due to methylation [8].

In FSHD2, the mutations that disrupt the open reading frame are believed to result in *SMCHD1* haploinsufficiency and to have less effect on D4Z4 methylation than mutations which maintain the *SMCHD1* open reading frame, possibly acting through a dominant negative mechanism [8]. These new developments are improving our understanding of the pathology of FSHD and emphasize the potential for combinatorial effects of genetic and epigenetic factors in influencing the onset and progression of the disease.

## Conclusions

In this study, we propose an improved (as compared to the previously described [25]) approach for predicting the possible location of remotely acting regulatory elements that might influence the transcriptional regulation of their associated gene(s). This approach was successfully employed in the context of the in silico prediction of potential remotely acting regulatory elements for the *SMCHD1* gene. Subsequent sequencing of these predicted regions identified three sequence variants in FSHD patients which represent candidate variants of potential functional significance.

This approach demonstrates a novel means to screen for disease-relevant mutations that reside beyond the immediate vicinity of a given disease gene. It therefore promises not only to be useful in investigating disorders in which mutations may occur in remotely acting regulatory elements but also in identifying the causative non-coding mutations found by GWAS that are often distant from their target genes.

## Methods

### Long-range interaction data (Hi-C)

In December 2013, when this study commenced, the inter- and intra-chromosomal interaction data were available for, respectively, 1-Mb and 100-kb fragments from the human GM06990 lymphoblastoid cell line. These data were obtained by means of the chromosome conformation capture technique [14] and were available at www.ncbi.nlm.nih.gov/geo/ (accession number GSE18199). Data, recently obtained using in situ Hi-C method [15] at a 1-kb resolution, together with Capture Hi-C data on looping interactions for ~22,000 promoters [16], were used to validate the initial results.

### Epigenetic features, transcription factor binding sites and enhancers

Data on the occurrence and the strength of DNaseI hypersensitive sites, the occurrence and level of enrichment in the H3k4me1, H3k4me3 and H3k27ac methylation marks, assayed using various biochemical techniques, were downloaded from the ENCODE database ([26]; http://genome.ucsc.edu/index.html).

Genomic positions of 55 transcription factor binding sites (TFBSs), including an insulator CTCF-binding site, all assayed by ChIP-seq, were also downloaded from the ENCODE database. Genomic positions of active enhancers assayed using CAGE techniques were downloaded from the FANTOM (http://fantom.gsc.riken.jp/5/; [27]) and ENCODE (ChromHMM track) databases. It should be noted that biochemical assays used to map these functional elements usually capture extended DNA regions, often spanning several hundred base pairs, making it difficult to define the exact boundaries of functional elements.

In cases where data were available from multiple cell lines, the data for the karyotypically normal human lymphoblastoid cell line GM06990 were used. The choice of this cell line was predicated upon the availability of Hi-C data for this particular cell line. Data on H3k4me1 and H3K27ac methylation marks were not available for the GM06990 cell line; data for the GM12878 cell line were used instead.

Where necessary, all positions were ‘lifted over’ to the hg18 assembly using the Lift Genome Annotation program available at https://genome.ucsc.edu/cgi-bin/hgLiftOver.

### In silico prediction of possible remotely acting regulatory regions and sites of mutation

To identify possible remotely acting regulatory regions and the sites of mutation within these regions, the following step-by-step procedure was adopted:

- Step 1. Set the size of the fragment to 1 Mb or 100 kb.
- Step 2. Use Hi-C data to find a fragment (bin) that harbours the gene/gene promoter in question, henceforth termed *a gene-containing fragment*.
- Step 3. Use Hi-C data to find a fragment(s) with the highest number of interactions with the gene-containing fragment, henceforth termed *an interacting fragment*(*s*).
- Step 4. Use ENCODE data to identify the location(s) of CTCF-binding sites within the interacting fragment. Refine the interacting fragment(s) to shorter regions bounded by the CTCF-binding site. If the interacting fragment is found upstream of the gene, a DNA fragment between the CTCF-binding site and the gene in question is considered as a region possibly harbouring remotely acting regulatory element(s). If the interacting fragment is found downstream of the gene, a DNA fragment between the gene and the CTCF-binding site is considered as a region possibly harbouring remotely acting regulatory element(s).
- Step 5. Use the ENCODE and FANTOM data on the occurrence of epigenetic marks, enhancers, etc., to further refine regions possibly harbouring remotely acting regulatory element(s) by identifying shorter regions enriched in histone modification marks (H3K4me1, H3K27ac, H3K4me3) and transcription factor binding sites and corresponding to peaks of DNaseI hypersensitivity signal.
- Step 6. Validate regions found using in situ and Capture Hi-C data.

### The *SMCHD1* gene and clinical data

The *SMCHD1* gene, encoding the structural maintenance of chromosomes’ flexible hinge domain-containing protein 1, occupies positions 2,645,885–2,795,015 on chromosome 18 (NCBI build 37, UCSC hg18 and Ensembl GRCh37).

Of the 229 FSHD patients analysed in this study, 29 had >11 D4Z4 repeat units whereas 2 of these patients harboured novel FSHD-causing (or disease-modifying) sequence variants in the *SMCHD1* gene identified in FSHD patients by Winston et al. [10] that could modulate the expression of FSHD1.

### DNA sequencing and methylation analysis

The DNA from patients was digested with *Eco*RI and *Bln*I, size-fractionated by electrophoresis, Southern blotted and hybridized with DNA marker p13E11 [1]; sequencing was performed as described in Additional file 1. Methylation quantification was performed as described by Hartweck et al. [21]; see Additional file 1 for details.

## Additional file

Additional file 1:
**Summary of experimental procedures used.** A brief description of DNA sequencing and methylation analysis used in this study.
