# Supplementary material for: Remotely acting SMCHD1 gene regulatory elements: in silico prediction and identification of potential regulatory variants in patients with FSHD
Source: Hum Genomics. 2015 Oct 7;9:25. doi: 10.1186/s40246-015-0047-x (PMC4597391; doi:10.1186/s40246-015-0047-x)
Supplement: Additional file 1: — Summary of experimental procedures used. A brief description of DNA sequencing and methylation analysis used in this study. [file 40246_2015_47_MOESM1_ESM.docx]

**A novel approach to *in silico* prediction of variants in remotely-acting gene regulatory elements**

**Additional file 1**

Mary B Mayes^1^, Daniel S Buxton^1^, Taniesha Morgan^2^, Jincy Winston^2^, Mihir A Kamat^1^, Rebecca L Martin^1^, Dirk A Kleinjan^3^, Meena Upadhyaya^2^, David N Cooper^2^ and Nadia Chuzhanova^1^

^1^School of Science and Technology, Nottingham Trent University, Nottingham NG11 8NS, UK.

^2^Institute of Medical Genetics, School of Medicine, Cardiff University, Heath Park, Cardiff CF14 4XN, UK.

^3^MRC Human Genetics Unit, Institute of Genetics and Molecular Medicine, University of Edinburgh, Edinburgh EH4 2XU, UK.

* Corresponding author: Nadia Chuzhanova, School of Science and Technology, Nottingham Trent University, Clifton Lane, Nottingham, NG11 8NS, UK. Tel: +44 (0)115 848 8304 E-mail: nadia.chuzhanova@ntu.ac.uk

*DNA sequencing*

The PCR mixture contained 7uL AmpliTaq Gold 360 Mastermix, 25ng genomic DNA and 1.6uL 2uM forward and reverse primers and distilled water to a final volume of 20uL. The PCR cycling parameters used with a DNA thermal cycler were as follows: hot start at 95°C for 10 mins, followed by 40 cycles denaturation at 94°C for 1 min, annealing at 60°C for 1 min and extension at 72°C for 1 min and a final extension at 72°C for 10 mins. Sequencing was performed with the BigDye® terminator v1.1 cycle sequencing kit (Applied Biosystems) and analysed on AB3730 DNA Analyser. Sequence analysis was performed using Sequencher (Gene Codes Corporation). Details of the PCR sequencing primers are given in Supplementary Table 1.

**Supplementary Table 1.** Details of the PCR sequencing primers**.**

|  | PCR primers 5’-3’ | Location of primers | Size of the PCR product |
| --- | --- | --- | --- |
| Region1_1a forward | CTTCTGACCTCGTGGTCCAC | hg18 assembly, chr18: 2631444-2631463 | 581bp |
| Region1_1a reverse | TGAGCTCCTTCTCAGATGGTAT | hg18 assembly, Chr18: 2632003 - 2632024 |  |
| Region 1_ 1b forward | TCTCTTGCTGGCAAGCATCA | hg18 assembly, Chr18: 2631851 - 2631870 | 426bp |
| Region 1_ 1b reverse | TCAACTTTCCTTGAAATCCTCTG | hg18 assembly, Chr18: 2632254 - 2632276 |  |
| Region 2 forward | GTTTCCAGGGCGAGTTGAG | hg18 assembly: chr18: 2561314-2561332 | 287bp |
| Region 2 reverse | CAAAACACCAGGTCCTTCCT | hg18 assembly: chr18: 2561581-2561600 |  |

*Methylation analysis*

Methylation quantification was performed on ten statistically validated sites in the first D4Z4 repeat that was found to exhibit extreme demethylation caused by FSHD2 (Hartweck et al. 2013). Bisulphite conversion was followed by pyrosequencing, and the ratio of C vs T at each variable site was averaged over the ten sites. The methylation threshold was set at ≤ 42%, with samples between 42 and 45% repeated.

.
